# Paternal high-fat diet altered SETD2 gene methylation in sperm of F0 and F1 mice

**DOI:** 10.1186/s12263-023-00731-4

**Published:** 2023-08-19

**Authors:** Suhua Wei, Shiwei Luo, Haifeng Zhang, Yandong Li, Juan Zhao

**Affiliations:** 1 Department of Hematology, The First Affiliated Hospital of Xi’an Jiaotong University, Xi’an, Shaanxi China; 2 State Key Laboratory of Oncology in South China, Sun Yat-Sen University, Guangzhou, China; 3 Xi’an International Medical Center Hospital, Xi’an, Shaanxi China

**Keywords:** Obesity, SETD2, Epigenetic, Sperm, High fat diet

## Abstract

Paternal high-fat diet (HFD) can alter the epigenetics of sperm DNA, resulting in the transmission of obesity-related traits to the offspring. Previous studies have mainly focused on the HFD-induced changes in DNA methylation of imprinted genes, overlooking the potential involvement of non-imprinted genes in this process. *SETD2*, an important epigenetically-regulated gene known for its response to environmental stress, remains poorly understood in the context of high-fat diet-induced epigenetic changes. Here we examined the effect of obesity from a HFD on paternal *SETD2* expression and methylation in sperm, and embryos at the blastocyst stage and during subsequent development, to determine the alteration of *SETD2* in paternal intergenerational and transgenerational inheritance. The result showed that mice fed with HFD for two months had significantly increased SETD2 expression in testis and sperm. The paternal HFD significantly altered the DNA methylation level with 20 of the 26 CpG sites being changed in sperm from F0 mice. Paternal high-fat diet increased apoptotic index and decreased total cell number of blastocysts, which were closely correlated with DNA methylation level of sperm. Out of the 26 CpG sites, we also found three CpG sites that were significantly changed in the sperm from F1 mice, which meant that the methylation changes at these three CpG sites were maintained.

In conclusion, we found that paternal exposure to an HFD disrupted the methylation pattern of SETD2 in the sperm of F0 mice and resulted in perturbed SETD2 expression. Furthermore, the paternal high-fat diet influenced embryo apoptosis and development, possibly through the SETD2 pathway. The altered methylation of SETD2 in sperm induced by paternal HFD partially persisted in the sperm of the F1 generation, highlighting the role of SETD2 as an epigenetic carrier for paternal intergenerational and transgenerational inheritance.

**Supplementary Information:**

The online version contains supplementary material available at 10.1186/s12263-023-00731-4.

## Introduction

More than one billion adults in the world are overweight, and obesity is a chronic metabolic disease associated with a high prevalence of hypertension, diabetes, osteoarthritis, cancer, and cardiovascular disease [1]. A high-fat diet (HFD) is a major cause of obesity, which can also have harmful effects on male reproductive functions, by decreasing sperm count and motility, reducing semen quality, increasing sperm DNA damage and impairing sperm acrosome reactions [2]. Obesity can also cause disorders in embryonic development and impaired health of offspring [3, 4].

Previous studies have shown that epigenetic changes in sperm caused by paternal obesity could be passed on to offspring, leading to a range of diseases including reproductive impairment and metabolic disorders, such as insulin resistance in both male and female offspring, hyperleptemia, and lipid accumulation in ovaries of female offspring [5–9]. Progeny of male mice fed a HFD also exhibited dysregulation of olfactory transduction pathways in fat and islet tissues, and beta cell dysfunction [10]. Epigenetic information carriers such as histone modifications, DNA methylation, miRNAs and tRNAs in sperm from obese mice were altered, and those changes were directly related to diseases in progeny [11, 12].

DNA methylation of imprinted genes is an important epigenetic information carrier that mediates parental inheritance. During nuclear reprogramming of early embryos, DNA methylation of imprinted genes largely maintains the original modification pattern [13, 14]. Small changes in the differentially methylated regions (DMR) of imprinted genes can cause serious damage to the health of offspring. For example, minor change in the DMR methylation of *IGF2*, an imprinted gene, could result in a doubling or halving of IGF2 transcription [15–17]. Whether this influence also exists for non-imprinted genes remains unclear. SETD2 is an important gene that is regulated epigenetically and plays a crucial role in responding to environmental stress [18]. It is the only histone transferase for H3K36me3 that also participates in molecular processes such as maintaining genome stability, chromatin conformation, and gene transcription initiation and elongation [19, 20]. Here we focused on the effect of a paternal HFD on *SETD2* in sperm, and offspring, to determine the role of *SETD2* in parental intergenerational and transgenerational inheritance.

## Methods

### Animals and reagents

Mice were maintained under controlled temperature (22 ℃ ± 1℃) and humidity conditions with a 12:12 h light: darkness cycle. Two groups of 4-week-old male ICR mice were randomly assigned to receive either a CD (Research Diets, D12450B) containing 10% of the kcal as fat or a HFD (Research Diets, D12492) containing 60% of the kcal as fat. After eight weeks of feeding, males from each group were then mated (1:1 ratio) with 12-week-old female estrus ICR mice fed the CD. The pregnancy rate of the CD or HFD group was the number of the pairs who gave birth divided by the number of mating pairs. All offspring mice were fed the CD. Euthanize mice using cervical dislocation. All procedures performed in mice were approved by the Laboratory Animal Care Committee of Xi’an Jiaotong University. Unless otherwise specified, all reagents are purchased from Sigma-Aldrich.

### Hematoxylin and eosin (H&E) staining and immunohistochemistry

Testes were fixed in modified Davidson’s fixative for 18 h, then in 10% formaldehyde solution for 24 h. The testis was cross-sectioned from the middle, and dehydrated with graded alcohol, cleared in xylene and embedded in wax, then cut into 4 µm sections. After dehydration and dewaxing, sections were mounted with neutral gum, and digitally imaged with a microscope (Olympus, Tokyo, Japan). For immunohistochemistry of SETD2, dewaxed testis sections were heated in a pressure cooker for 30 min in EDTA buffer, then endogenous peroxide activity was quenched with 3% H_2_O_2_ for 15 min_._ The slides were blocked with 10% BSA for 30 min, and incubated with the SETD2 primary antibody (Abclonal, 1:500 dilution, Wuhan, China) overnight at 4℃. The slides were washed with PBS, incubated with enhanced enzyme-labeled goat anti-rabbit IgG (Abclonal) for 1 h at room temperature (RT), and then visualized using a DAB substrate kit (ZSGB Biotech, Beijing, China) counterstained with hematoxylin. Images were captured using a light microscope (Olympus).

### Collection of sperm and embryos

The bilateral vas deferens and epididymis were dissected, and placed in a 2 mL of Dulbecco’s phosphate-buffered saline (DPBS, Univ, Shanghai, China). The vas deferens and epididymal capsule were cut open with ophthalmic scissors, incubated at 37℃ for 10 min, and the tissue fragments were separated from the sperm, which were then collected by centrifugation at 3,000 × g for 10 min at 4℃. The supernatants were discarded and the pellets were suspended in 1 mL of DPBS. One mL of 50% Percoll was placed in a 15 mL centrifuge tube, and 1 mL of washed sperm was added (1 mL of 50% Percoll for approximately every 100 million spermatozoa). Tubes were placed on ice for 10 min, then centrifuged at 800 × g for 20 min at 4℃. The 50% Percoll layer was discarded and the sperm was recovered. The sperm was washed with 2 mL of DPBS and centrifuged at 3,000 × g for 5 min at 4℃. The supernatants were carefully removed and discarded, and then collected the sperms. Sperm was counted using a sperm quality testing system (XD-6000X, XINDA, Xuzhou, China).

Twelve-week-old female ICR mice were injected with 5 IU of serum gonadotrophin (NSHF, Ningbo, China), followed by the injection of 5 IU of chorionic gonadotrophin after two days to induce superovulation, and then mated with males from the CD or HFD group. Next morning, the female mice with vaginal plugs were used for collecting embryos. Embryos at the blastocyst stage were collected from the uterus and the method is described as follows. Using ophthalmic forceps, grasp the cervix and cut it with ophthalmic scissors. Gently lift the uterus with the forceps and cut the uterine ligaments with the scissors until reaching the uterine tip, then cut between the fallopian tubes and ovaries to extract the uterine horn. In a glass dish containing flushing fluid, use ophthalmic scissors to remove any accessory structures attached to the uterine horn, and rinse it thoroughly to recover the embryos. During embryo flushing, place the uterine horn on a flat dish and use ophthalmic scissors to longitudinally cut open the junction of the uterine tube. Using a syringe filled with flushing fluid, insert the needle into the cervical opening and flush the uterine cavity, allowing the embryos to be washed out with the fluid flow, using about 1.0 mL of liquid on each side. Apoptotic index in blastocysts and total cell number (TCN) in blastocysts were counted as previous report [21].

### RNA extraction, cDNA synthesis and qPCR

Total RNA was extracted from the 50 mg (weighed with electronic analytical balance, Beyotime, Shanghai, China; E0241) sperm pellets with 1 mL of TRIzol by repeated pipetting for 5 min and vortexing for 30 s. Beta-mercaptoethanol (40 μL) was added, mixed well, and tubes were incubated at 65℃ for 45 min, then immediately placed on ice for 1 min. Chloroform (200 μL) was added, the tubes were shaken for 15 s, then allowed to stand for 10 min at RT. Tubes were centrifuged at 12,000 × g for 15 min at 4℃. The aqueous phase was removed, mixed with 500 μL of isopropanol, kept at 4℃ for 15 min, then centrifuged at 12,000 × g for 10 min at 4℃. The supernatants were discarded and the pelleted RNA was washed with 500 μL of 75% ethanol and 500 μL of absolute ethanol, and centrifuged at 5000 × g for 5 min at 4℃. Supernatants were discarded and the pellets were allowed to dry at RT. The RNA was dissolved in 20 μL of RNase-free water. Reverse transcription to cDNA and qPCR were performed as previously described [21, 22]. Details of the primers are described in Table S1.

### Western blotting

Total protein was extracted from sperm (50 mg) using a lysis buffer containing 7 M urea, 2 M thiourea, 1% CHAPS, 1% n-octyl-glucopyranoside, 0.5% IPG buffer, 18 mM DTT, and 2.4 mM PMSF. Sperm pellets were suspended in lysis buffer and gently shaken for 1 h at RT, then centrifuged at 3,000 × g for 5 min at 4℃. The supernatants containing the solubilized sperm proteins were recovered. RIPA buffer was used to extract proteins from embryos, and lysates were centrifuged at 3,000 × g for 5 min at 4℃ to recover the proteins. Protein aliquots were separated by SDS–polyacrylamide gel electrophoresis and transferred to polyvinylidene difluoride membranes. The membranes were blocked with 5% nonfat milk powder in Tris-buffered saline containing 0.05% Tween-20 (TBST) for 1 h, and then incubated with SETD2 primary antibody (Abclonal, 1:500 dilution) for 24 h at 4℃, followed by thorough washing in TBST. The blots were then incubated with secondary antibody (Abclonal) for 1 h at RT and proteins were detected using an enhanced chemiluminescence kit (Millipore, Billerica, MA, USA).

### Methylation analysis

Bisulfite conversion of DNA samples (500 ng) was done using the EZ DNA methylation kit (Zymo Research, Irvine, CA, USA). The CpG sites were tested by pyrosequencing. Specific primers were designed for CpG loci at the *SETD2* promoter region using PyroMark software (Qiagen, Hilden, Germany). The PCR product was sequenced using PyroMark Q48 (Qiagen). The methylation level for the target region was quantified using the PyroQ-CpG software (Qiagen).

### Statistical analysis

Body weight, relative level of mRNA and protein, methylation level, and total cell numbers in blastocysts were determined and compared by unpaired Student’s *t* test using Graph Pad Prism (version 9; Graph Pad Inc.; San Diego, CA, USA). A *P* < 0.05 was considered statistically significant. All the data are presented as mean ± SEM.

## Results

### F0 mice fed a HFD exhibited abnormal *SETD2* expression in sperm

**Fig. 1:**
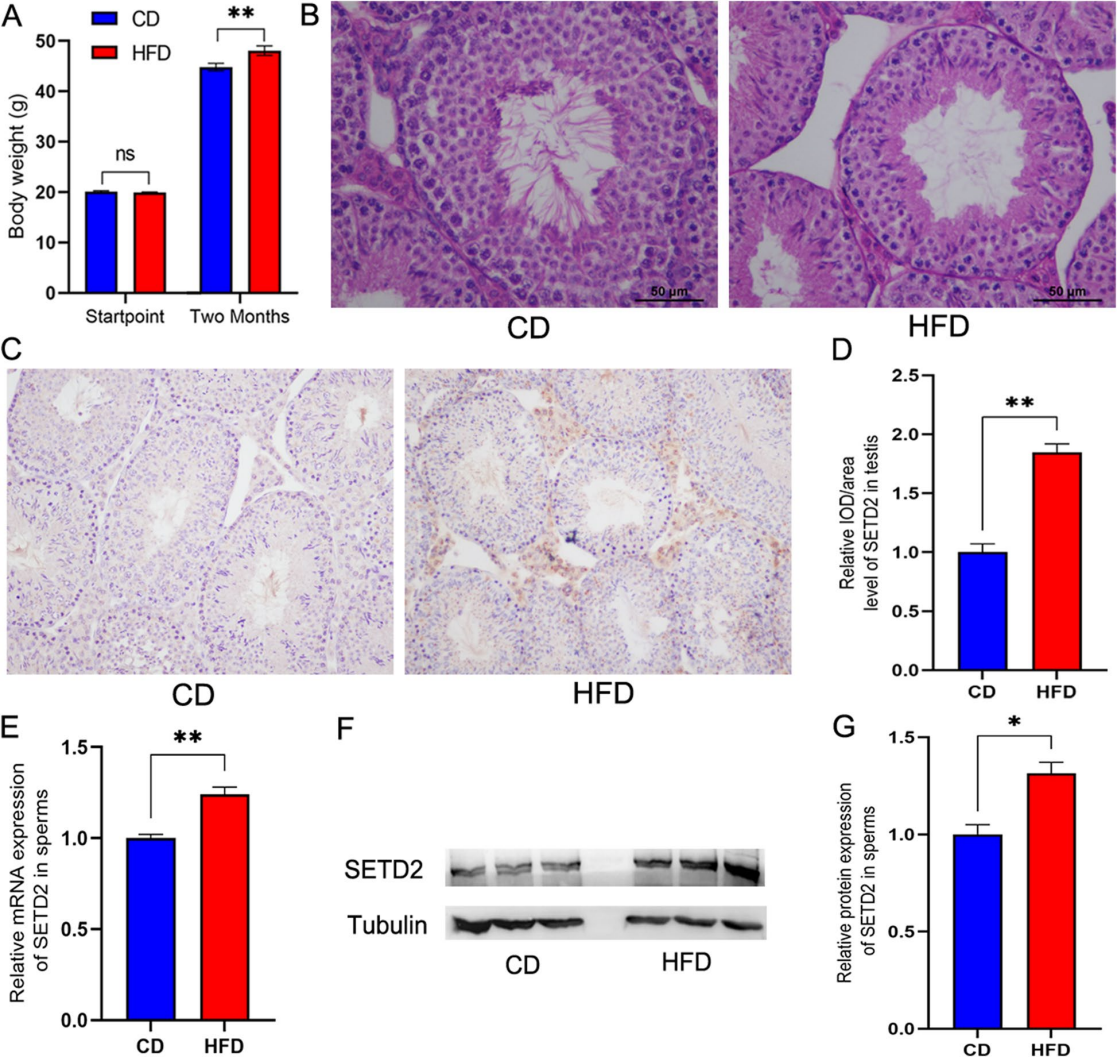
Effect of HFD on SETD2 expression in testis and sperm of F0 mice. **A** Significant weight gain of F0 male mice fed HFD over two months. **B** Morphological analysis of F0 mice testis in CD group (*n* = 10) and HFD group (*n* = 10) with H&E staining. **C** Detection of SETD2 in F0 mice testis from the two groups (*n* = 10 in each group) by immunohistochemistry, and **D** the relative level of SETD2 expression in F0 mouse testis in the two group. **E** Relative expression of SETD2 mRNA in F0 mouse sperm determined by qPCR (*n* = 10 in each group). **F** Determination of SETD2 protein from F0 mouse sperm by western blotting (*n* = 3 in each group), and **G** relative expression level of SETD2 protein in F0 mouse sperm from the two group (*n* = 10 in each group). ** above the bars indicates *P* < 0.01, and * above the bars indicates *P* < 0.05

F0 mice were fed a HFD or normal diet (CD) for two months, and the HFD group (*n* = 20) had significantly higher body weight than the CD group (*n* = 20) (Fig. 1A). Testicular tissue from CD mice was closely arranged and the seminiferous tubules were neatly arranged, while in the HFD group, the seminiferous tubules were loosely arranged and the number of spermatogenic cells was lower (Fig. 1B). Immunohistochemistry showed that SETD2 level in the testis was significantly higher in the HFD group than in the CD group (Fig. 1C&D). Sperm was extracted and subjected to qPCR and western blotting, and the expression of SETD2 in the HFD group was significantly higher than that in the CD group (Fig. 1E, F&G).

### F0 mice fed a HFD showed abnormal methylation of the *SETD2* promoter region in sperm DNA

**Fig. 2:**
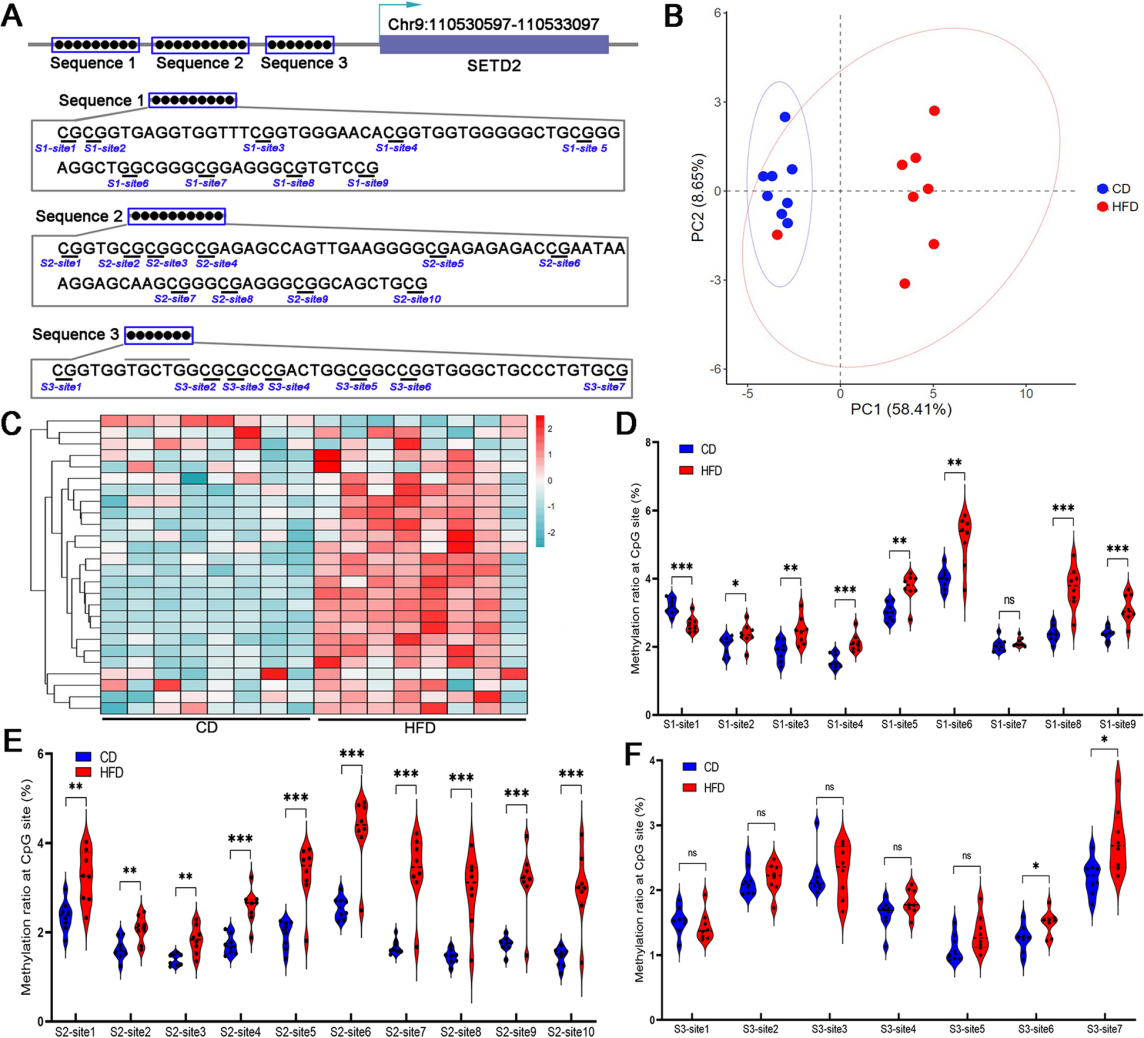
Paternal HFD altered methylation profile of SETD2 promoter region in sperm from F0 mice. **A** Genomic structure and relative position of the region sequenced are shown, and a total of 26 sites with at least three CpG sequences in the SETD2 promoter region were selected for methylation profiling. The numbering of the CpGs below the sequence corresponds to each of the CpGs analyzed. The methylation level of the 26 sites was measured by pyrosequencing followed by principal component analysis (**B**) and heatmap analysis (**C**). The methylation levels of the two groups are shown for Seq1 (**D**), Seq2 (**E**) and Seq3 (**F**). *** above the bars indicates *P* < 0.001, ** above the bars indicates *P* < 0.01, and * above the bars indicates *P* < 0.05

Sperm from the two groups (8 male mice in each group) was extracted, and 26 sites with at least three CpG-rich sequences (Seq1, Seq2, Seq3) in the *SETD2* promoter region were selected for determination of methylation level by pyrosequencing. Seq1 contained nine CpG sites (S1, sites 1- 9); Seq2 contained ten CpGs sites (S2, sites1-10); and Seq3 contained seven CpG sites (S3, sites 1–7) (Fig. 2A). Methylation level determination followed by principal component analysis (PCA) and heatmap construction showed significant differences in methylation levels in sperm DNA from F0 mice fed a HFD compared to CD (Fig. 2B, C). Analysis of Seq1 showed that S1-site1 was significantly lower in the HFD group than in the CD group, while S1-sites 2–6, 8 & 9 were significantly higher with HFD than CD (Fig. 2D). Methylation of CpG sites at Seq2 was significantly higher with HFD than CD (Fig. 2E). No significant difference in methylation at S3-sites 1–5 was found between HFD and CD mice, while it was significantly higher at S3-sites 6 and 7 in HFD (Fig. 2F). HFD altered DNA methylation in the promoter region of *SETD2* in sperm DNA; 20 of the 26 CpG sites were significantly changed in the HFD group compared to the CD group.

### Apoptotic index and TCN in blastocysts were closely related with the methylation level of SETD2 in sperms

**Fig. 3:**
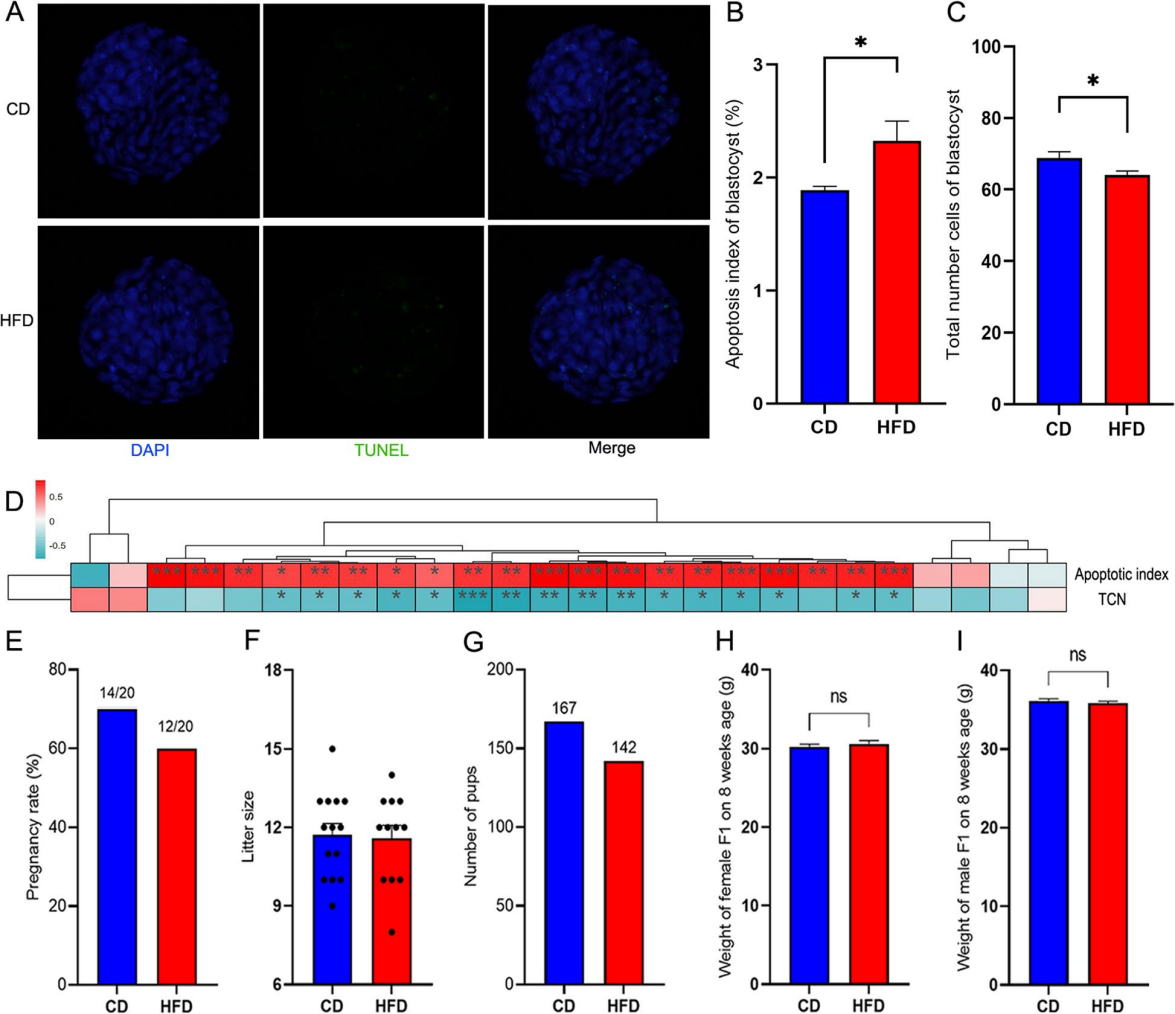
Paternal HFD reduced total cell numbers in blastocysts. Representative images of blastocysts in each group showing TUNEL assay results for apoptotic cells (green). Apoptotic index (**B**), and total cell numbers (**C**) in blastocysts fertilized by F0 mouse sperm between the two groups. **D** Correlations between the methylation level of SETD2, apoptotic index, and total cell numbers in blastocysts. Spearman’s correlation coefficients are represented by colors ranging from blue (−1) to red (+1). Pregnancy rate (**E**), litter size (**F**), and number of pups born (**G**) between the two groups is shown. Body weights of the F1 males (**H**) and females (**I**) from the two groups. *** above the bars indicates *P* < 0.001, ** above the bars indicates *P* < 0.01, and * above the bars indicates *P* < 0.05

F0 male mice from the CD and HFD groups were mated with female CD mice, then the apoptotic index and TCN in blastocysts was analyzed. The apoptotic index in blastocysts from the HFD group was significantly higher than from the CD group, and the TCN in blastocysts from the HFD group was significantly lower than from the CD group (Fig. 3A-C). Also we found that the apoptotic index and total cell numbers in blastocysts were closely related with the methylation level of SETD2 in sperms of F0 (*n* = 8 in each group) (Fig. 3D). To evaluate the pregnancy rate, F1 litter size, total numbers and weights of the pups, 40 adult female CD mice weighing 26–28 g in estrus were caged (1 male + 1 female) with male CD mice (*n* = 20) or HFD mice (*n* = 20) for one day. As a result of the mating, 14 female mice in the CD group and 12 female mice in the HFD group gave birth (Fig. 3E). There was no significant difference in the average litter size between the two groups (Fig. 3F). The total number of pups from the HFD mouse mating was lower than from the CD group (142 vs 167, Fig. 3G). After feeding with CD for two months, the body weights of the F1 mice from the two groups were not significantly different (Fig. 3G, I).

### Paternal HFD altered methylation profile of *SETD2* promoter region in F1 sperm

**Fig. 4:**
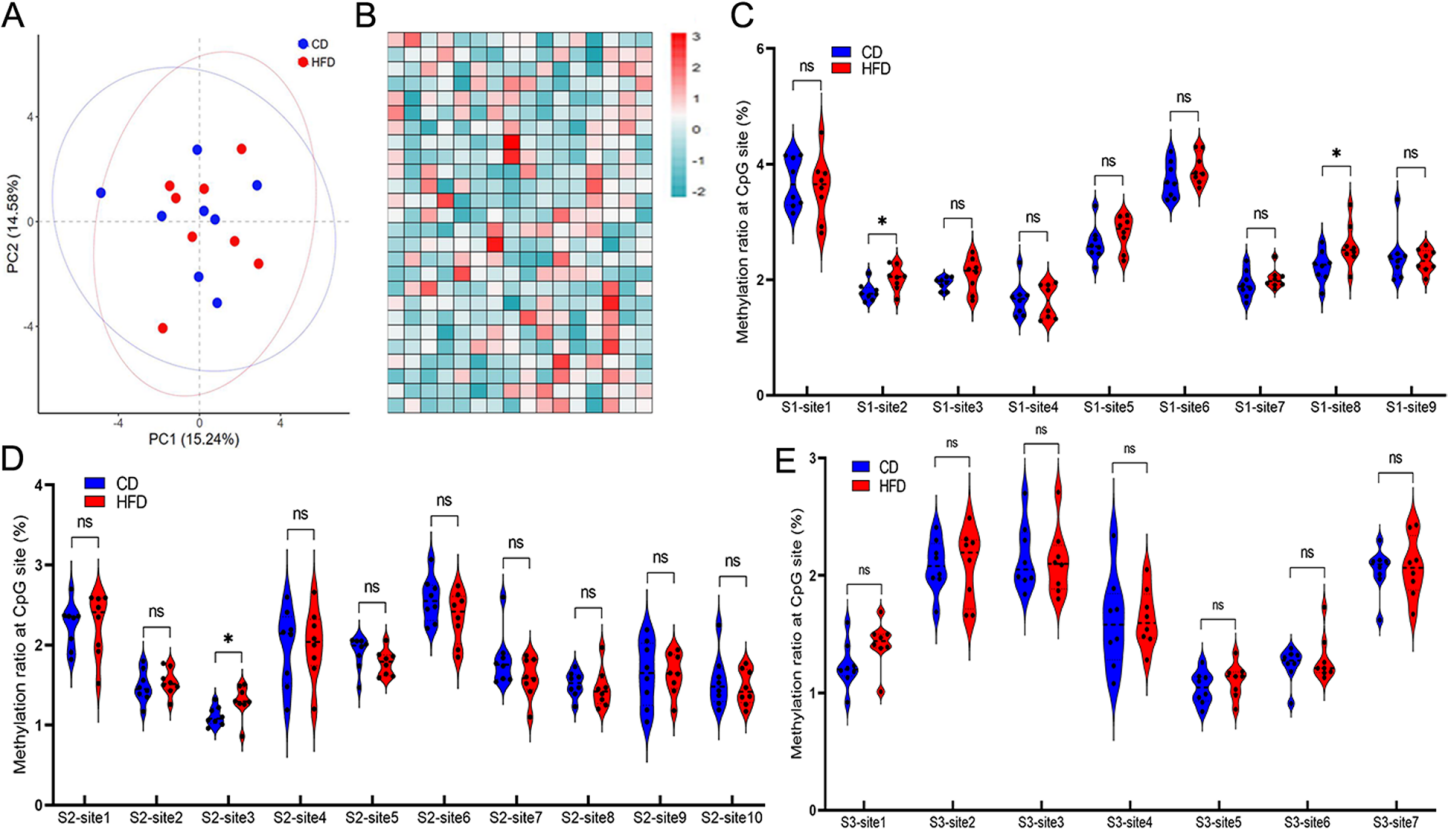
Paternal HFD altered methylation profile of SETD2 promoter region in F1 sperm. Methylation levels of the 26 sites in F1 sperm by pyrosequencing followed by principal component analysis (**A**) and heatmap analysis (**B**). Methylation level of Seq1 (**C**), Seq2 (**D**) and Seq3 (**E**) F1 sperm between the two groups. * above the bars indicates *P* < 0.05

Sperm was extracted from F1 male mice (8 male mice in each group), and the DNA methylation levels at the 26 sites were measured. The PCA and heatmap profiles were similar between the two groups (Fig. 4A, B). The methylation level at 23 of the 26 CpG sites was not significantly different between the two groups (Fig. 4C, D&E); however, the CpG sites, S1-site2, S1-site8, and S2-site3 were significantly higher in the HFD group than the CD group, which seemed to maintain the changes seen in the sperm DNA of F0 mice (Fig. 4C, D).

## Discussion

The *SETD2*-H3K36me3 axis plays an important role in genetic mutation and epigenetics, which is an important regulatory system for organisms to respond to environmental stress [18]. *SETD2* knockout in mice led to abnormal embryonic vascular remodeling and death [23]. Here we found abnormal expression of SETD2 in the testis of mice fed a HFD. A previous study reported that a HFD damages the intestinal barrier so that toxic metabolites can enter the body from the intestine, cross the blood-testis barrier, and cause inflammation [24]. Activation of *SETD2* by inflammatory stimulation could mediate H3K36me3, thus promoting stimulus–response transcription [25]. Here, the abnormal expression of *SETD2* in the testis might be attributable to inflammation induced by HFD.

Paternal stress can affect offspring through histone modification, DNA methylation, imprinted gene methylation and increased content of non-coding RNAs in sperm [12, 26, 27]. In fact, sperm contains only a very small amount of histone and mRNA, and most sperm DNA methylation undergoes reprogramming after fertilization [28]. Whether *SETD2* is involved in intergenerational or transgenerational inheritance as an important gene regulating epigenetics and responding to environmental stress has not been reported. In this study, we showed for the first time that a HFD could significantly alter the mRNA and protein expression of sperm *SETD2*, and also change the DNA methylation pattern in the *SETD2* promoter.

In order to explore whether HFD alteration of DNA methylation at the *SETD2* promoter region in F0 sperm could be passed on to F1 mice through the sperm, we measured the DNA methylation level at 26 CpG sites in sperm DNA from 2-month-old F1 mice, and found three CpG sites in the F1 sperm DNA that retained some of paternal HFD-induced changes as in the sperm of F0 mice. This result indicates that a paternal HFD can not only alter the *SETD2* methylation pattern of F0 sperm and the level of H3K36me3 in embryos, but also affect *SETD2* methylation in F1 sperm, suggesting that the effects of a HFD in male mice is traceable in the sperm epigenome. Although the paternal HFD-induced epigenetic memory patterns in the sperm *SETD2* methylation profile were significantly different in F0 mice between the two groups at 20 of the 26 CpG sites, 17 of the 20 changed CpG sites did not differ significantly in HFD vs CD F1 mice, indicating that the epigenetic memory induced by paternal HFD could be partially lost in the offspring.

Some studies demonstrated that paternal exposure to malnutrition, such as high fat, low protein, or low folate or environmental toxicants, impaired sperm function and altered DNA methylation patterns [29–31]. Paternal exposure to cigarette smoke increased the global methylation of sperm DNA and altered the DMR of the imprinted gene, DLK1, in the F1 generation, which may be inherited and may perturb long-term metabolic function [32]. Recent studies found that sepsis impaired sperm function and altered the DNA methylome, causing disruptions of immune responses in male offspring [33, 34]. Most of the reported studies on sperm DNA methylation have mainly used non-targeted detection of DNA methylation, or targeted detection of methylation of individual imprinted genes, with very little focus on non-imprinted genes [35–38]. In this study, pyrosequencing was used to detect the DNA methylation of *SETD2* in sperm, and revealed that paternal HFD-induced epigenetic changes in the *SETD2* methylation pattern could be transferred to the F1 generation. This discovery opens a window onto new opportunities for uncovering environmental factors mediating sperm epigenetics from a new perspective. Some past studies posited that gene methylation was negatively correlated with transcriptional silencing, and CpG methylation of enhancer/promoter sequences could abolish specific factor binding as well as transcriptional activation, but this conclusion was not absolutely true [39–41]. High-throughput sequencing methods have examined the effect of partial gene methylation on transcription factor binding and found that about one-third of the gene methylation sites were preferentially favored by transcription factors [41]. In the present study, we found that the methylation level of the sperm DNA at the *SETD2* promoter region was negatively correlated with the expression level of *SETD2*, which also was consistent with the hypothesis that gene methylation was not always negatively correlated with gene expression.

*SETD2*/H3K36me3 plays a crucial role in regulating cell apoptosis and chromatin accessibility [42]. When cells suffer severe DNA damage, *SETD2* is activated and localized near the broken DNA, catalyzing H3K36me3, thereby regulating DNA damage repair and maintaining genomic stability [43]. Apoptosis-related genes such as FAS and P53 were targeted by *SETD2*/H3K36me3 [44]. Mutations in *SETD2* can lead to increased genomic instability, hinder DNA damage repair, and disrupt apoptotic pathways [42–44]. Here, we found that a paternal high-fat diet significantly altered the methylation levels and expression of the *SETD2* promoter region in sperm, which may be related to the metabolic disorder in the testes induced by a high-fat diet. Here, our results showed that a paternal high-fat diet increased the apoptotic index and decreased the TCN in blastocysts, and a positive correlation between *SETD2* methylation levels and blastocyst apoptotic rate, and a negative correlation with the TCN in blastocysts, indicating that a paternal high-fat diet mediate embryo apoptosis through sperm *SETD2* regulation.

In conclusion, we found that male F0 mice fed a HFD showed abnormal *SETD2* expression, as well as an abnormal methylation pattern of the *SETD2* promoter region in sperm. We also showed that paternal HFD affected the *SETD2* methylation pattern of F1 sperm, suggesting that dietary changes in F0 male mice fed a HFD were traceable in the sperm epigenome as *SETD2* methylation patterns in F1 offspring.

### Supplementary Information

**Additional file 1: Table S1.** Primers of qPCR.

**Additional file 2: Tables S2-S4.** Primers of the Sequence1-3 of SETD2 for Methylation analysis.

**Additional file 3: Table S5-S7.** Methylation value of each site of the Sequence1-3 of SETD2 in the F0 sperms between the CD and HFD group.

**Additional file 4: Tables S8-S10.** Methylation value of each site of the Sequence1-3 of SETD2 in the F1 sperms between the CD and HFD group.

## Data Availability

Datasets analyzed during the current study will be made available from the corresponding author upon reasonable request. The supplementary data is available as additional files in the manuscript.
