# Supplementary material for: Paternal high-fat diet altered SETD2 gene methylation in sperm of F0 and F1 mice
Source: Genes Nutr. 2023 Aug 19;18:12. doi: 10.1186/s12263-023-00731-4 (PMC10439541; doi:10.1186/s12263-023-00731-4)
Supplement: Supplementary file 1 — Additional file 1: Table S1. Primers of qPCR. [file 12263_2023_731_MOESM1_ESM.docx]

**Table S1. Primers of qPCR**

| Gene | Sequence | Nt (bp) | Product size (bp) | Tm(ºC) |
| --- | --- | --- | --- | --- |
| SETD2 | F: TTTGCCCAAAGGCACGAAGA | 20 | 114 | 60 |
|  | R: AGCGCAGTGAGAAATCTATTCTG | 23 |  |  |
| β-actin | F: GTACCACCATGTACCCAGGC | 20 | 247 | 60 |
|  | R: AACGCAGCTCAGTAACAGTCC | 21 |  |  |
| Ppia | F: GAGCTCTGAGCACTGGAGAGA | 21 | 85 | 60 |
|  | R: CCACCCTGGCACATGAAT | 18 |  |  |
