# Supplementary material for: Paternal high-fat diet altered SETD2 gene methylation in sperm of F0 and F1 mice
Source: Genes Nutr. 2023 Aug 19;18:12. doi: 10.1186/s12263-023-00731-4 (PMC10439541; doi:10.1186/s12263-023-00731-4)
Supplement: Supplementary file 2 — Additional file 2: Tables S2-S4. Primers of the Sequence1-3 of SETD2 for Methylation analysis. [file 12263_2023_731_MOESM2_ESM.docx]

**Table S2. Primers of the Sequence1 of SETD2 for Methylation analysis**

| Primer | Sequence | Nt (bp) | Tm(ºC) | %GC |
| --- | --- | --- | --- | --- |
| PCR-F | TGATTTTTAGGGAGAGGGTAGAATAT | 26 | 56.9 | 34.6 |
| PCR-R | CACCCAAAACCAAACTAAACAAAAACTACT | 30 | 59.0 | 33.3 |
| Sequencing | ACTTCTCAACCTAAACTT | 18 | 45.2 | 33.3 |
| Sequence to Analyze | CTCRAACACR CCCTCCRCCC RCCAACCTCC CRCAACCCCC ACCACCRTAT TCCCACCRAA ACCACCTCAC CRCRATATTC TACCCTC | | | |
| Amplicon length | 145 | | | |

**Table S3. Primers of the Sequence2 of SETD2 for Methylation analysis**

| Primer | Sequence | Nt (bp) | Tm(ºC) | %GC |
| --- | --- | --- | --- | --- |
| PCR-F | GAGAAGTTTAGGTTGAGAAGTAGTTT | 26 | 59.5 | 34.6 |
| PCR-R | TCAAAACACCCCCCTTCATCCTTC | 24 | 57.8 | 50.0 |
| Sequencing | AGTTTTTGTTTAGTTTGGT | 19 | 44.6 | 26.3 |
| Sequence to Analyze | TTTYGGTGYG YGGTYGAGAG TTAGTTGAAG GGGYGAGAGA GATYGAATAA AGGAGTAAGY GGGYGAGGGY GGTAGTTGYG TTATTTTAGT GTT | | | |
| Amplicon length | 183 | | | |

**Table S4. Primers of the Sequence3 of SETD2 for Methylation analysis**

| Primer | Sequence | Nt (bp) | Tm(ºC) | %GC |
| --- | --- | --- | --- | --- |
| PCR-F | GGAAGTTGTTTAGGGAGGGGTAG | 23 | 59.7 | 52.2 |
| PCR-R | CCTTACAAACCCAACCCCTTAAA | 23 | 60.9 | 43.5 |
| Sequencing | GTTGTATTTAGTTTTTGTTAGGGT | 24 | 45.9 | 29.2 |
| Sequence to Analyze | YGGTGGTGTT GGYGYGTYGA TTGGYGGTYG GTGGGTTGTT TTGTGYGTGT TTTTTTAAGG GGTTGGGTTT GT | | | |
| Amplicon length | 220 | | | |
