# Supplementary material for: Paternal high-fat diet altered SETD2 gene methylation in sperm of F0 and F1 mice
Source: Genes Nutr. 2023 Aug 19;18:12. doi: 10.1186/s12263-023-00731-4 (PMC10439541; doi:10.1186/s12263-023-00731-4)
Supplement: Supplementary file 3 — Additional file 3: Table S5-S7. Methylation value of each site of the Sequence1-3 of SETD2 in the F0 sperms between the CD and HFD group. [file 12263_2023_731_MOESM3_ESM.docx]

**Table S5. Methylation value (%) of each site of the Sequence1 of SETD2 in the F0 sperms between the CD and HFD group**

| Sample(F0) | S1-site1 | S1-site2 | S1-site3 | S1-site4 | S1-site5 | S1-site6 | S1-site7 | S1-site8 | S1-site9 |
| --- | --- | --- | --- | --- | --- | --- | --- | --- | --- |
| CD1 | 3.32 | 1.67 | 1.90 | 1.83 | 3.39 | 3.53 | 2.14 | 2.01 | 2.41 |
| CD2 | 3.21 | 1.64 | 1.94 | 1.41 | 2.86 | 3.98 | 2.12 | 2.35 | 2.67 |
| CD3 | 3.05 | 2.19 | 2.25 | 1.43 | 3.01 | 4.01 | 1.91 | 2.20 | 2.17 |
| CD4 | 3.43 | 2.24 | 1.56 | 1.52 | 3.35 | 3.68 | 1.85 | 2.46 | 2.35 |
| CD5 | 3.49 | 2.20 | 2.03 | 1.76 | 3.01 | 3.83 | 2.02 | 2.63 | 2.41 |
| CD6 | 3.04 | 1.86 | 1.73 | 1.88 | 3.15 | 4.11 | 2.01 | 2.78 | 2.37 |
| CD7 | 2.83 | 2.33 | 2.22 | 1.48 | 2.76 | 4.54 | 2.45 | 2.25 | 2.56 |
| CD8 | 3.00 | 2.11 | 1.42 | 1.30 | 2.69 | 4.21 | 1.88 | 2.38 | 2.08 |
| HFD1 | 2.69 | 2.29 | 2.00 | 2.11 | 3.65 | 4.39 | 2.25 | 3.93 | 2.88 |
| HFD2 | 2.53 | 2.49 | 2.49 | 1.91 | 4.00 | 5.69 | 2.07 | 4.20 | 3.09 |
| HFD3 | 2.41 | 2.28 | 2.44 | 2.28 | 3.64 | 5.85 | 2.04 | 3.33 | 3.50 |
| HFD4 | 2.74 | 2.57 | 2.82 | 2.14 | 3.70 | 5.34 | 2.05 | 4.00 | 3.68 |
| HFD5 | 2.44 | 2.25 | 2.21 | 1.85 | 4.15 | 5.43 | 2.07 | 3.44 | 2.96 |
| HFD6 | 2.81 | 2.40 | 3.21 | 2.68 | 4.02 | 5.61 | 2.01 | 4.68 | 3.53 |
| HFD7 | 2.55 | 2.88 | 2.51 | 2.03 | 3.92 | 5.05 | 2.17 | 3.65 | 2.97 |
| HFD8 | 3.13 | 1.75 | 2.08 | 1.95 | 2.80 | 3.66 | 2.39 | 2.64 | 2.45 |

**Table S6. Methylation value (%) of each site of the Sequence2 of SETD2 in the F0 sperms between the CD and HFD group**

| Sample(F0) | S2-site1 | S2-site2 | S2-site3 | S2-site4 | S2-site5 | S2-site6 | S2-site7 | S2-site8 | S2-site9 | S2-site10 |
| --- | --- | --- | --- | --- | --- | --- | --- | --- | --- | --- |
| CD1 | 2.31 | 1.24 | 1.31 | 1.72 | 1.98 | 2.40 | 1.50 | 1.34 | 1.74 | 1.28 |
| CD2 | 2.21 | 1.94 | 1.55 | 2.07 | 2.20 | 2.71 | 1.72 | 1.18 | 1.66 | 1.54 |
| CD3 | 2.45 | 1.96 | 1.23 | 1.77 | 1.76 | 2.25 | 1.57 | 1.49 | 1.77 | 1.46 |
| CD4 | 2.10 | 1.52 | 1.28 | 1.55 | 2.25 | 2.68 | 1.61 | 1.47 | 1.85 | 1.71 |
| CD5 | 1.81 | 1.54 | 1.49 | 2.00 | 1.41 | 2.43 | 1.80 | 1.70 | 1.79 | 1.57 |
| CD6 | 2.45 | 1.61 | 1.50 | 1.52 | 2.11 | 2.71 | 1.57 | 1.36 | 1.99 | 1.59 |
| CD7 | 2.96 | 1.70 | 1.27 | 1.47 | 2.22 | 2.27 | 2.01 | 1.61 | 1.65 | 1.08 |
| CD8 | 2.59 | 1.47 | 1.48 | 1.65 | 1.62 | 2.96 | 1.59 | 1.49 | 1.38 | 1.25 |
| HFD1 | 2.77 | 1.69 | 2.29 | 2.73 | 3.06 | 4.49 | 4.03 | 3.25 | 3.03 | 3.01 |
| HFD2 | 3.85 | 2.16 | 1.79 | 2.74 | 3.62 | 4.32 | 3.86 | 2.26 | 3.24 | 2.60 |
| HFD3 | 2.74 | 2.38 | 1.52 | 2.44 | 3.37 | 4.28 | 3.25 | 3.39 | 3.37 | 2.99 |
| HFD4 | 4.03 | 2.46 | 1.95 | 3.23 | 3.16 | 4.80 | 3.60 | 2.98 | 3.52 | 2.91 |
| HFD5 | 3.61 | 2.04 | 1.69 | 2.65 | 3.66 | 4.91 | 4.22 | 3.95 | 4.15 | 4.19 |
| HFD6 | 3.27 | 2.16 | 2.18 | 2.34 | 3.86 | 4.13 | 3.12 | 3.47 | 3.13 | 3.09 |
| HFD7 | 3.25 | 2.04 | 1.88 | 2.66 | 3.82 | 4.85 | 3.32 | 2.83 | 3.19 | 3.65 |
| HFD8 | 2.32 | 1.61 | 1.35 | 1.88 | 1.81 | 2.49 | 1.67 | 1.37 | 1.48 | 1.32 |

**Table S7. Methylation value (%) of each site of the Sequence3 of SETD2 in the F0 sperms between the CD and HFD group**

| Sample(F0) | S3-site1 | S3-site2 | S3-site3 | S3-site4 | S3-site5 | S3-site6 | S3-site7 |
| --- | --- | --- | --- | --- | --- | --- | --- |
| CD1 | 1.55 | 2.39 | 2.30 | 1.72 | 0.94 | 0.93 | 2.66 |
| CD2 | 1.49 | 1.95 | 1.97 | 1.71 | 1.50 | 1.24 | 2.33 |
| CD3 | 1.73 | 2.56 | 2.20 | 1.77 | 0.95 | 1.42 | 2.12 |
| CD4 | 1.56 | 1.96 | 2.13 | 1.13 | 0.92 | 1.59 | 2.33 |
| CD5 | 1.38 | 2.13 | 2.08 | 1.67 | 1.31 | 1.29 | 2.09 |
| CD6 | 1.85 | 1.94 | 3.04 | 1.91 | 1.21 | 1.27 | 2.35 |
| CD7 | 1.15 | 2.15 | 2.03 | 1.52 | 0.98 | 1.28 | 1.78 |
| CD8 | 1.52 | 2.05 | 2.07 | 1.58 | 1.06 | 1.05 | 1.95 |
| HFD1 | 1.24 | 2.35 | 2.58 | 1.73 | 1.87 | 1.54 | 3.69 |
| HFD2 | 1.58 | 2.25 | 1.84 | 2.01 | 1.16 | 1.53 | 2.73 |
| HFD3 | 1.39 | 2.09 | 2.75 | 1.77 | 1.11 | 1.50 | 2.39 |
| HFD4 | 1.93 | 2.37 | 2.70 | 2.09 | 1.00 | 1.55 | 2.90 |
| HFD5 | 1.29 | 2.48 | 2.04 | 1.69 | 1.42 | 1.81 | 2.64 |
| HFD6 | 1.48 | 1.73 | 1.67 | 1.99 | 1.57 | 1.25 | 3.20 |
| HFD7 | 1.26 | 2.20 | 2.30 | 1.78 | 1.32 | 1.58 | 2.34 |
| HFD8 | 1.36 | 2.05 | 2.42 | 1.50 | 1.21 | 1.22 | 2.22 |
