# Supplementary material for: Paternal high-fat diet altered SETD2 gene methylation in sperm of F0 and F1 mice
Source: Genes Nutr. 2023 Aug 19;18:12. doi: 10.1186/s12263-023-00731-4 (PMC10439541; doi:10.1186/s12263-023-00731-4)
Supplement: Supplementary file 4 — Additional file 4: Tables S8-S10. Methylation value of each site of the Sequence1-3 of SETD2 in the F1 sperms between the CD and HFD group. [file 12263_2023_731_MOESM4_ESM.docx]

**Table S8. Methylation value (%) of each site of the Sequence1 of SETD2 in the F1 sperms between the CD and HFD group**

| Sample(F1) | S1-site1 | S1-site2 | S1-site3 | S1-site4 | S1-site5 | S1-site6 | S1-site7 | S1-site8 | S1-site9 |
| --- | --- | --- | --- | --- | --- | --- | --- | --- | --- |
| CD1 | 3.28 | 1.65 | 2.05 | 1.38 | 2.59 | 4.05 | 2.00 | 2.65 | 2.40 |
| CD2 | 4.15 | 1.82 | 1.78 | 1.46 | 2.45 | 3.47 | 1.83 | 2.49 | 2.04 |
| CD3 | 3.33 | 1.61 | 1.99 | 1.73 | 2.47 | 4.22 | 2.33 | 2.05 | 2.45 |
| CD4 | 3.43 | 1.88 | 1.78 | 1.35 | 2.21 | 3.91 | 1.60 | 2.09 | 2.41 |
| CD5 | 4.16 | 2.11 | 2.05 | 2.30 | 2.77 | 3.66 | 2.16 | 2.29 | 2.32 |
| CD6 | 3.87 | 1.72 | 1.90 | 1.74 | 2.56 | 3.69 | 1.85 | 1.76 | 2.00 |
| CD7 | 4.11 | 1.75 | 1.95 | 1.64 | 3.28 | 3.40 | 1.91 | 2.24 | 2.22 |
| CD8 | 3.15 | 1.76 | 1.99 | 1.70 | 2.70 | 3.38 | 1.71 | 2.28 | 3.39 |
| HFD1 | 2.92 | 2.06 | 2.48 | 1.29 | 3.00 | 4.29 | 1.84 | 2.55 | 2.41 |
| HFD2 | 2.81 | 1.66 | 2.21 | 1.95 | 3.10 | 3.59 | 1.94 | 2.40 | 2.23 |
| HFD3 | 4.55 | 2.30 | 2.18 | 1.91 | 2.82 | 3.78 | 1.91 | 3.30 | 2.24 |
| HFD4 | 3.72 | 2.04 | 1.64 | 1.91 | 2.94 | 3.83 | 1.92 | 2.45 | 2.18 |
| HFD5 | 3.43 | 1.94 | 2.13 | 1.82 | 3.12 | 4.30 | 2.40 | 2.92 | 2.60 |
| HFD6 | 3.84 | 2.28 | 2.36 | 1.46 | 2.33 | 3.69 | 2.08 | 2.58 | 2.47 |
| HFD7 | 3.59 | 2.07 | 1.71 | 1.36 | 2.42 | 4.05 | 2.01 | 2.48 | 2.52 |
| HFD8 | 3.87 | 1.86 | 1.94 | 1.32 | 2.73 | 3.85 | 2.06 | 2.05 | 2.01 |

**Table S9. Methylation(%) value of each site of the Sequence2 of SETD2 in the F1 sperms between the CD and HFD group**

| Sample(F1) | S2-site1 | S2-site2 | S2-site3 | S2-site4 | S2-site5 | S2-site6 | S2-site7 | S2-site8 | S2-site9 | S2-site10 |
| --- | --- | --- | --- | --- | --- | --- | --- | --- | --- | --- |
| CD1 | 2.08 | 1.35 | 1.05 | 2.41 | 2.06 | 2.76 | 1.54 | 1.39 | 2.19 | 1.74 |
| CD2 | 2.34 | 1.39 | 1.32 | 1.65 | 1.47 | 3.07 | 1.57 | 1.47 | 1.04 | 1.19 |
| CD3 | 2.36 | 1.47 | 1.01 | 1.19 | 1.98 | 2.45 | 1.90 | 1.58 | 1.72 | 1.27 |
| CD4 | 2.70 | 1.56 | 1.10 | 2.19 | 1.87 | 2.71 | 1.77 | 1.23 | 1.41 | 1.37 |
| CD5 | 2.36 | 1.17 | 1.06 | 1.48 | 2.05 | 2.21 | 1.84 | 1.45 | 1.94 | 1.53 |
| CD6 | 1.91 | 1.71 | 1.22 | 2.16 | 1.74 | 2.26 | 1.74 | 1.73 | 1.19 | 1.43 |
| CD7 | 1.82 | 1.43 | 1.18 | 2.13 | 2.01 | 2.48 | 2.60 | 1.64 | 2.05 | 1.60 |
| CD8 | 2.37 | 1.80 | 0.96 | 2.60 | 2.05 | 2.62 | 1.58 | 1.60 | 1.58 | 2.25 |
| HFD1 | 2.35 | 1.74 | 1.29 | 1.84 | 1.75 | 2.63 | 1.42 | 1.39 | 1.43 | 1.77 |
| HFD2 | 1.92 | 1.43 | 1.41 | 2.00 | 1.59 | 1.94 | 1.52 | 1.25 | 1.18 | 1.47 |
| HFD3 | 2.59 | 1.26 | 1.51 | 1.71 | 1.88 | 2.50 | 1.87 | 1.62 | 1.64 | 1.35 |
| HFD4 | 2.00 | 1.50 | 1.26 | 1.20 | 1.61 | 2.74 | 1.57 | 1.20 | 1.53 | 1.17 |
| HFD5 | 1.52 | 1.53 | 0.86 | 2.35 | 1.64 | 1.85 | 1.81 | 1.32 | 1.65 | 1.66 |
| HFD6 | 2.58 | 1.58 | 1.49 | 2.24 | 1.86 | 2.55 | 1.10 | 1.97 | 1.85 | 1.36 |
| HFD7 | 2.47 | 1.48 | 1.27 | 2.66 | 2.06 | 2.34 | 1.60 | 1.47 | 1.94 | 1.71 |
| HFD8 | 2.59 | 1.77 | 1.30 | 2.08 | 1.83 | 2.24 | 1.82 | 1.45 | 1.89 | 1.26 |

**Table S10. Methylation (%) value of each site of the Sequence3 of SETD2 in the F1 sperms between the CD and HFD group**

| Sample(F1) | S3-site1 | S3-site2 | S3-site3 | S3-site4 | S3-site5 | S3-site6 | S3-site7 |
| --- | --- | --- | --- | --- | --- | --- | --- |
| CD1 | 1.40 | 2.15 | 1.84 | 1.89 | 1.13 | 1.38 | 2.13 |
| CD2 | 1.13 | 1.98 | 2.39 | 1.71 | 0.96 | 1.18 | 2.01 |
| CD3 | 1.18 | 2.41 | 2.70 | 1.70 | 1.26 | 1.33 | 2.12 |
| CD4 | 1.23 | 2.19 | 2.11 | 2.34 | 1.10 | 1.32 | 1.62 |
| CD5 | 1.21 | 1.69 | 1.99 | 1.08 | 0.84 | 1.25 | 1.97 |
| CD6 | 0.92 | 2.30 | 1.96 | 1.43 | 0.92 | 1.25 | 2.07 |
| CD7 | 1.19 | 2.01 | 2.30 | 1.46 | 1.14 | 1.30 | 2.30 |
| CD8 | 1.60 | 1.97 | 1.98 | 1.23 | 0.99 | 0.91 | 2.12 |
| HFD1 | 1.50 | 1.88 | 2.16 | 1.46 | 1.03 | 1.17 | 1.85 |
| HFD2 | 1.69 | 2.31 | 2.11 | 1.65 | 0.98 | 1.43 | 2.41 |
| HFD3 | 1.39 | 1.66 | 2.09 | 1.48 | 1.34 | 1.21 | 1.95 |
| HFD4 | 1.41 | 2.13 | 1.93 | 1.54 | 1.18 | 1.21 | 1.67 |
| HFD5 | 1.01 | 1.66 | 1.87 | 2.05 | 1.17 | 1.26 | 2.11 |
| HFD6 | 1.47 | 2.26 | 2.71 | 1.73 | 1.14 | 1.73 | 2.43 |
| HFD7 | 1.38 | 2.49 | 1.80 | 1.88 | 1.14 | 1.18 | 2.13 |
| HFD8 | 1.50 | 2.28 | 2.29 | 1.28 | 0.86 | 1.13 | 2.02 |
